# CDC Grand Rounds: Promoting Hearing Health Across the Lifespan

**DOI:** 10.15585/mmwr.mm6708a2

**Published:** 2018-03-02

**Authors:** William J. Murphy, John Eichwald, Deanna K. Meinke, Shelly Chadha, John Iskander

**Affiliations:** ^1^National Institute for Occupational Safety and Health, CDC; ^2^National Center for Environmental Health, CDC; ^3^University of Northern Colorado, Dept. of Audiology and Speech Sciences, Greeley, Colorado; ^4^World Health Organization, Geneva, Switzerland; ^5^Office of the Associate Director for Science, CDC.

Globally, one in three adults has some level of measurable hearing loss, and 1.1 billion young persons are at risk for hearing loss attributable to noise exposure. Although noisy occupations such as construction, mining, and manufacturing are primary causes of hearing loss in adults, nonoccupational noise also can damage hearing. Loud noises can cause permanent hearing loss through metabolic exhaustion or mechanical destruction of the sensory cells within the cochlea. Some of the sounds of daily life, including those made by lawn mowers, recreational vehicles, power tools, and music, might play a role in the decline in hearing health. Hearing loss as a disability largely depends on a person’s communication needs and how hearing loss affects the ability to function in a job. The loss of critical middle and high frequencies can significantly impair communication in hearing-critical jobs (e.g., law enforcement and air traffic control).

## Occupational Noise-Induced Hearing Loss

A recent analysis of 2011–2012 National Health and Nutrition Examination Survey (NHANES) data estimates that approximately 14% of U.S. adults aged 20–69 years (27.7 million persons) have hearing loss. After adjustments for age and sex, hearing impairment was nearly twice as prevalent in men as in women; age, sex, ethnicity, and firearm use were all important risk factors for hearing loss (*1*).

CDC’s National Institute for Occupational Safety and Health (NIOSH) estimates that 22 million workers are exposed to hazardous levels of noise in their workplaces (*2*). The estimated prevalence of hearing loss among noise-exposed workers is 12%–25%, depending on type of industry. Reductions in workplace noise and increased use of hearing protection might have contributed to a decreased prevalence of hearing loss over time in some sectors, including agriculture, forestry, fishing, and hunting and transportation, warehousing, and utilities (*3*). The risk for incident hearing loss (i.e., the likelihood of observing a new case of hearing loss in a worker’s longitudinal audiometric data) decreased by 46% from the periods 1986–1990 to 2006–2010 (*3*).

For high exposure levels such as firearm or aircraft noise above 140 decibels sound pressure level (dB SPL), engineering and administrative controls might not reduce noise exposures adequately. Such situations require hearing protection devices (HPDs) providing upwards of 30–40 dB of noise reduction when worn properly. Despite the existence of occupational regulations for hearing protection, many workers fail to achieve adequate protection because their earplugs or earmuffs do not fit properly. Hearing protector fit testing provides an opportunity to train workers to properly fit hearing protectors and to encourage effective use. The NIOSH HPD Well-Fit hearing protector fit-test system is a simple, portable solution for testing in quiet office spaces. Other fit-testing systems are commercially available (*4*).

## Nonoccupational Noise-Induced Hearing Loss

Primary sources of nonoccupational hearing loss in the United States include noise exposure from recreational hunting or shooting, use of personal music players, overexposure at concerts and clubs, and certain hobbies (e.g., motorsports and woodworking with power tools). In 2016, CDC began initiatives to raise awareness about the risk for permanent hearing damage attributable to nonoccupational noise exposures, including the development of new communication tools about noise-induced hearing loss. An analysis of 2011–2012 NHANES audiometric data from 3,583 adults aged 20–69 years identified persons with high-frequency audiometric notches suggestive of noise-induced hearing loss (*5*). Persons with normal hearing can detect sounds equally soft at all frequencies. When hearing is damaged by noise, the hearing test will show a loss of acuity in a narrow range of middle to high frequencies (3–6 kHz) with better hearing at both lower and higher frequencies. Often, the earliest sign is a notched configuration in the audiogram (Figure).

**FIGURE:**
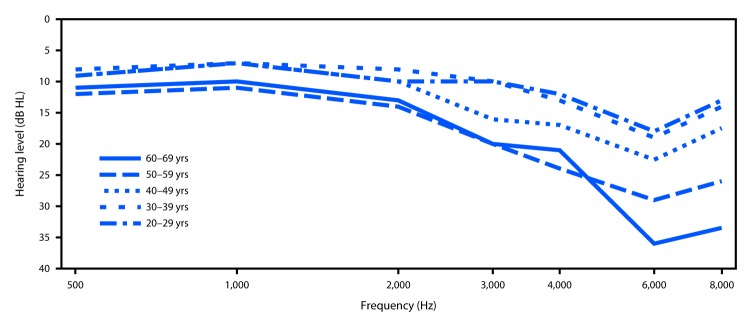
Mean audiometric thresholds for persons aged 20–69 years with identified unilateral (right ear only) notches — National Health and Nutrition Examination Survey, United States 2011–2012

The weighted prevalence of an audiometric notch was 24%, extrapolated to represent nearly 40 million U.S. adults. Unilateral audiometric notches were three times more prevalent than were bilateral audiometric notches and were more prevalent in men than in women. Participants who reported having exposure to loud noise at work were twice as likely to have evidence of hearing damage as were those who did not. However, 20% of persons with no occupational exposure to loud noise had an audiometric notch, suggesting that 21 million U.S. adults likely have hearing damage from noise at home or in their communities (*5*). The presence of an audiometric notch increased with age, ranging from 19% of participants aged 20–29 years to 29% of those aged 40–49 years. The prevalence of notches decreased among persons aged 50–59 years, as high-frequency hearing loss associated with aging increasingly masks the notch associated with noise-induced hearing loss (Figure).

Regardless of whether participants’ exposure was to work or recreational noise, 24% of those with such damage reported that their hearing was excellent or good, suggesting that many persons might be either unaware of or ignoring noise-induced hearing damage. Although most noise-induced hearing loss is preventable, the NHANES analysis found that 70% of persons exposed to loud noise in the past 12 months had seldom or never worn hearing protection (*5*).

Noise-induced hearing loss in youths is not a new problem. An analysis of 1988–1994 NHANES data identified audiometric notches in 20% of males and 12% of females aged 12–19 years among a population of 5,249 U.S. children and young adults aged 6–19 years (*6*). An analysis of 2005 and 2006 NHANES data found that 17% of both males and females had notched audiograms (*7*).

## Hearing Loss Worldwide

Hearing loss affects tens of millions of persons in the United States and hundreds of millions of persons worldwide, and during the past few decades, the estimated number of persons with hearing loss has steadily increased (*8*). The World Health Organization (WHO) estimates that approximately 360 million persons live with disabling hearing loss, including approximately 328 million (91%) adults (56% males and 44% females) and 32 million (9%) children. As the population ages, it is estimated that approximately 320 million persons aged >65 years will have hearing loss by 2030 and approximately 500 million by 2050 (*8*).

## National Prevention Efforts

To ensure that all persons can benefit from efforts to prevent noise-induced hearing loss, a coordinated public health hearing loss reduction and mitigation approach should focus on effective population-based preventive interventions that go beyond clinical service and traditional areas of diagnosis, treatment, and research and focus on epidemiologic surveillance, health promotion, and disease prevention. Such an approach can help determine the needs of the population and the barriers to care, leading to policies for prevention and management of hearing loss. Health communication science provides a theoretical framework to study, develop, and evaluate interventions designed to change individual behavior. Some of these theories have been applied in the promotion of hearing health.

Dangerous Decibels (http://dangerousdecibels.org/) is an evidence-based intervention program that has changed knowledge, attitudes, beliefs, and behaviors of both youths and adults for the prevention of noise-induced hearing loss and tinnitus. The messaging incorporates three strategies for hearing loss prevention: 1) turn it down, 2) walk away, and 3) protect your ears. Originally developed for youths, Dangerous Decibels has been successfully adapted for civilian adults and the military, and its effectiveness was demonstrated in randomized trials among children in the United States and in studies in New Zealand and Brazil (*9*,*10*). Comparison of responses to predelivery and two postdelivery questionnaires found that participants in the Dangerous Decibels presentation exhibited substantial improvements in knowledge, attitudes, and intended behaviors related to hearing and hearing loss prevention that were partially maintained 3 months after the presentation. Most recently, Dangerous Decibels expanded into a community-based intervention and is self-sustaining in U.S. Native American communities (*9*). The materials are in use in all 50 states, four U.S. territories, and 41 countries. Online games and activities are available, including Jolene, a system that measures music-listening sound levels and aids in educational outreach for hearing health (*11*).

CDC has developed tools and communication products to promote best practices for hearing loss prevention. In addition to practical engineering controls, administrative controls, and using hearing protectors, NIOSH promotes the Buy Quiet and Quiet-by-Design programs, designed for employers to take an inventory of their potentially harmful loud tools and replace them with quieter ones. Approximately 20 companies and individuals have been recognized for successful efforts by the Safe-in-Sound Excellence in Hearing Loss Prevention and Innovation Award (http://www.safeinsound.us/) developed by NIOSH and the National Hearing Conservation Association (*12*). In 2015, United Technologies, a corporation that serves customers in the commercial aerospace, defense, and building industries, received the award for promoting a hearing-loss prevention culture throughout the corporation. United Technologies reduced the number of persons exposed to hazardous noise by approximately 80%, thereby eliminating the need for a hearing conservation program for approximately 10,000 workers.

Other efforts include the promotion of recommended noise exposure standards for the workplace. NIOSH recommends an 85-dB limit for an average daily 8-hour exposure and a 3-dB exchange rate, which means that each increase of 3 dB in exposure level reduces the recommended exposure time by half (*13*). Thus, an 88-dB exposure limit is recommended for up to 4 hours and a 91-dB exposure limit for 2 hours. The National Hearing Conservation Association 85-3 Coalition, an organization of worker, professional, and industrial hygiene associations, promotes the use of an 85-dB limit and 3-dB exchange rate to protect the hearing of workers (*14*).

WHO focuses on undertaking evidence-based advocacy to raise awareness of deafness, hearing loss, and hearing care within all levels of society. WHO develops policy that advocates for hearing care provisions in its 194 member countries and develops standardized technical tools, recommendations, guidelines, and training resources to support policy development and implementation. It also engages directly with national ministries of health and other stakeholders to develop, implement, and monitor strategies for ear and hearing care.

Two principal advocacy initiatives promoted by WHO include World Hearing Day (http://www.who.int/pbd/deafness/world-hearing-day/en/) and the Make Listening Safe initiative (http://www.who.int/pbd/deafness/activities/MLS/en/) (*15*). The Make Listening Safe initiative was launched in 2015 to reduce the growing risk for hearing loss posed by unsafe listening practices in recreational settings. As part of this initiative, WHO is working with partners to develop technical standards and applications for personal audio systems and to promote safe listening practices among application (app) users. World Hearing Day, observed each year on March 3, aims to increase hearing loss awareness among policymakers, professionals, and communities. The 2018 theme is “Hear the future,” drawing attention to the globally increasing number of persons with hearing loss, focusing on preventive strategies, and outlining steps to ensure access to necessary rehabilitation services and communication tools and products.

Noise reduction and avoidance can prevent hearing loss or slow its progression. Persons can protect themselves by moving away or taking breaks from loud sounds, using quieter consumer products, lowering volumes on personal listening devices, reducing time listening to loud levels of music, and using hearing protectors. Hearing protectors need to fit well to reduce noise exposures effectively. Health care providers can inform patients about hearing loss symptoms, early diagnosis of hearing loss, and prevention strategies.

Policymakers, governments, and manufacturers of equipment can develop policies to reduce noise levels and limit noise exposures of the public. In parts of Europe, community noise and the effect of urban soundscapes on public health have received considerable attention. In the United States, national, state, and local community noise-control efforts are largely uncoordinated, potentially resulting in higher levels of community noise. Increasing awareness and reducing needless exposures to loud noise might help the public take appropriate steps to protect their hearing.
